# The lncRNA *TUG1* modulates proliferation in trophoblast cells via epigenetic suppression of *RND3*

**DOI:** 10.1038/cddis.2017.503

**Published:** 2017-10-12

**Authors:** Yetao Xu, Zhiping Ge, Erbao Zhang, Qing Zuo, Shiyun Huang, Nana Yang, Dan Wu, Yuanyuan Zhang, Yanzi Chen, Haoqin Xu, Huan Huang, Zhiyan Jiang, Lizhou Sun

**Affiliations:** 1 Department of Obstetrics and Gynecology, The First Affiliated Hospital of Nanjing Medical University, Nanjing, Jiangsu, China; 2 Department of Epidemiology and Biostatistics, Jiangsu Key Lab of Cancer Biomarkers, Prevention and Treatment, Collaborative Innovation Center for Cancer Personalized Medicine, School of Public Health, Nanjing Medical University, Nanjing, China; 3 Department of Emergency, The First Affiliated Hospital of Nanjing Medical University, Nanjing, Jiangsu, China; 4 The Family Planning Science and Technology Research Institute, Nanjing, Jiangsu, China

## Abstract

Due to limited treatment options, pre-eclampsia (PE) is associated with fetal perinatal and maternal morbidity and mortality. During the causes of PE, failure of uterine spiral artery remodeling which might be related to functioning abnormally of trophoblast cells, result in the occurrence and progression of PE. Recently, abnormal expression of long non-coding RNAs (lncRNAs), as imperative regulators involved in human diseases progression (included PE), which has been indicated by increasing evidence. In this research, we found that *TUG1*, a lncRNA, was markedly reduced in placental samples from patients with PE. Loss-function assays indicated that knockdown *TUG1* significantly affected cell proliferation, apoptosis, migration and network formation *in vitro*. RNA-seq revealed that *TUG1* could affect abundant genes, and then explore the function and regulatory mechanism of *TUG1* in trophoblast cells. Furthermore, RNA immunoprecipitation and chromatin immunoprecipitation assays validated that *TUG1* can epigenetically inhibit the level of *RND3* through binding to EZH2, thus promoting PE development. Therefore, via illuminating the *TUG1* mechanisms underlying PE development and progression, our findings might furnish a prospective therapeutic strategy for PE intervention.

Pre-eclampsia (PE), which is characterized by new-onset hypertension and proteinuria, is a pregnancy-specific syndrome.^1^ It is the major trigger of fetal morbidity and pregnancy-induced mortality, and it affects 3–5% of pregnancies worldwide, including in developing countries such as China.^2, 3^

In spite of the recent improvements made in treating PE, its underlying mechanism remains poorly understood. Previous studies have reported that many factors are involved in the pathogenesis of PE, such as oxygen dysregulation, impaired spiral artery remodeling and inappropriate maternal vascular destruction.^4, 5^ In PE, poor spiral artery remodeling is associated with dysfunctions of extravillous trophoblasts. For example, reduced proliferation,^6^ induced apoptosis^7^ and distorted migration and invasion abilities in extravillous trophoblasts^8^ may prevent them from successfully invading the myometrial spiral arteries.

Over the past decades, despite a focus on the function of protein-coding genes that participate in the pathogenesis of various diseases, long non-coding RNAs (lncRNAs), have received an increasing amount of attention with the development of whole-genome sequencing technologies.^9^ LncRNAs are RNA molecules longer than 200 nucleotides that do not encode proteins. Among the thousands of lncRNAs that have been revealed by the ENCODE project, only a few have been shown to be endowed with biological functions.^10, 11^ These lncRNAs are involved in a series of cellular progressions, such as parental imprinting, cell proliferation, apoptosis and metastasis via epigenetic modification, chromatin remodeling and sponging miRNAs.^12, 13, 14^ Recently, various researches have established that abnormal lncRNA levels might be associated with diverse human diseases, including PE. Previous studies have confirmed that abnormal lncRNAs may affect the proliferation, apoptosis and metastasis of trophoblast cells and stimulate the pathological placental development of PE.^15, 16^ These resulting data suggest that lncRNAs might have vital roles in the occurrence and progression of PE. Thus, to clarify the connection between PE-associated lncRNAs and their biological functions, a deeper understanding of the molecular mechanism of PE is essential.

Given the importance of lncRNAs in PE, in the present study, we focused on *TUG1*, a 7598-bp lncRNA gene located on chromosome 22q12.2. We found that the expression of *TUG1* is reduced in PE placental tissues compared with levels in controls. Moreover, loss-function assays were conducted to explore the influences of *TUG1* in the occurrence and development of PE. We found that *TUG1* might result in the impairment of spiral artery remodeling in PE. An experiment was conducted to establish the molecular mechanism by which *TUG1* modulates its targets in trophoblast cells. Our results provide a novel understanding of the biological functions of *TUG1* and the molecular regulatory mechanisms of its targets in trophoblasts.

## Results

### Deregulated expression of the *TUG1* in PE

**Figure 1:**
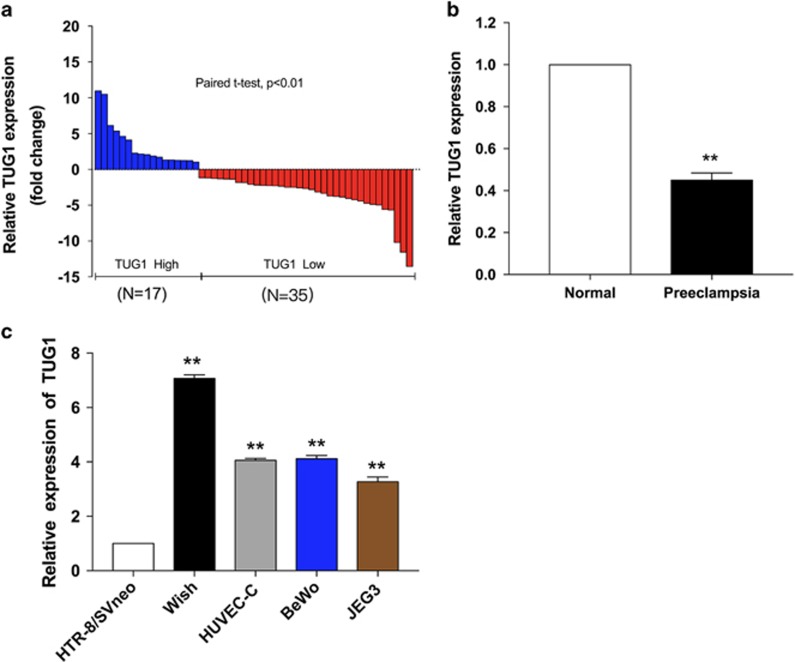
Relative *TUG1* expression in PE. (**a**) The relative expression of *TUG1* was measured by qRT-PCR. The levels of *TUG1* were lower in pre-eclamptic placentas samples (*N*=52) than in normal placentas (*N*=52). (**b**) Results are presented as the fold-change in PE placental samples relative to the control, and *TUG1* expression was classified into two groups. (**c**) *TUG1* expression were detected by qRT-PCR in several cell lines and were normalized to that in HTR-8/SVneo. At least three times of biological replicates have been performed and presented (values are mean±S.E.M.; **P<0.01)

We first detected *TUG1* expression levels in the placental tissues of a cohort of 52 pairs of PE patients and controls using qRT-PCR. There were no significant differences between the PE cases and controls in terms of gestation weeks or maternal age (*P*>0.05). Table 1 details the patient's clinical features. In particular, the levels of *TUG1* were significantly lower in PE placental samples compared with levels that in control tissues (Figures 1a and b).

### Effect of *TUG1* on the proliferation, migration and invasion in trophoblast cells

To explore the latent biological function of *TUG1* in trophoblast cells, we first assessed the levels of *TUG1* in several associated cell lines, such as HTR-8/SVneo, JEG-3, BeWo, WISH and HUVEC-C. We found that the expression of *TUG1* in HTR-8/SVneo and JEG-3 cells was lower than in the other cell lines (Figure 1c). Then we knocked down *TUG1* expression levels through the transfection of *TUG1* siRNAs in HTR-8/SVneo, JEG-3 and HUVEC-C cells. qRT-PCR analysis demonstrated that *TUG1* expression was silenced in si-*TUG1*-1#-treated cells and by si-*TUG1*-2#-treated cells (Supplementary Figure 1a).

**Figure 2:**
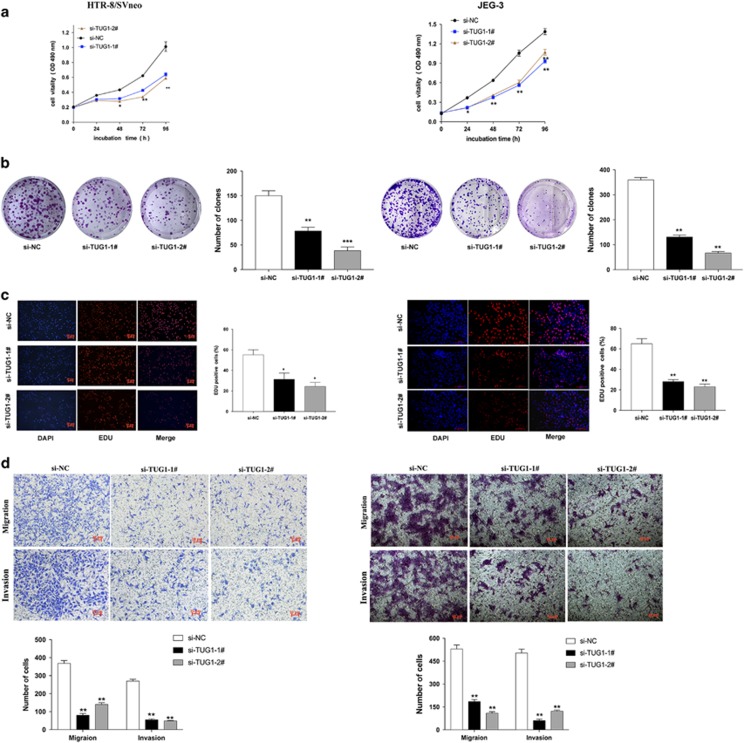
Effect of *TUG1* on proliferation, migration and invasion in trophoblast cells. (**a**) MTT assays were used to determine the viability of si-*TUG1*-transfected trophoblast cells. (**b**) Colony formation assays were performed to determine the proliferation of si-*TUG1*-transfected HTR-8/SVneo and JEG-3. Colonies were counted and captured. (**c**) Proliferating trophoblast cells were labeled with Edu. The Click-it reaction revealed Edu staining (red). Cell nuclei were stained with DAPI (blue). (**d**) The migration and invasion capacity of the cells transfected with si-*TUG1* was significantly lower than that of the negative control and higher in the cells overexpressing *TUG1*, as determined by transwell assays. All experiments were performed in biological triplicates with three technical replicates (values are mean±S.E.M.; **P<0.01, *P<0.05)

We next conducted an MTT assays that showed that cell proliferation was suppressed when *TUG1* levels were silenced in HTR-8/SVneo and JEG-3 cells (Figure 2a). In addition, a colony formation assay was performed, and the resulting data revealed that growth ability was reduced after downregulating *TUG1* in HTR-8/SVneo and JEG-3 (Figure 2b). Moreover, EdU staining assay also showed that knockdown of *TUG1* reduced the proliferation of these two types of cells (Figure 2c).

Because cell migration and invasion are essential features of PE progression, we performed transwell assays to examine the roles of *TUG1* on the migration or/and invasion abilities of HTR-8/SVneo and JEG-3 cells. Knockdown of *TUG1* inhibited the migration and invasion abilities, and the number of cells was significantly reduced compared with that in controls in HTR-8/SVneo and JEG-3 cells (Figure 2d). These results indicate that the knockdown of *TUG1* expression inhibits the invasion and migration phenotype in trophoblast cells.

### Effect of *TUG1* on cell cycle and apoptosis in trophoblasts

**Figure 3:**
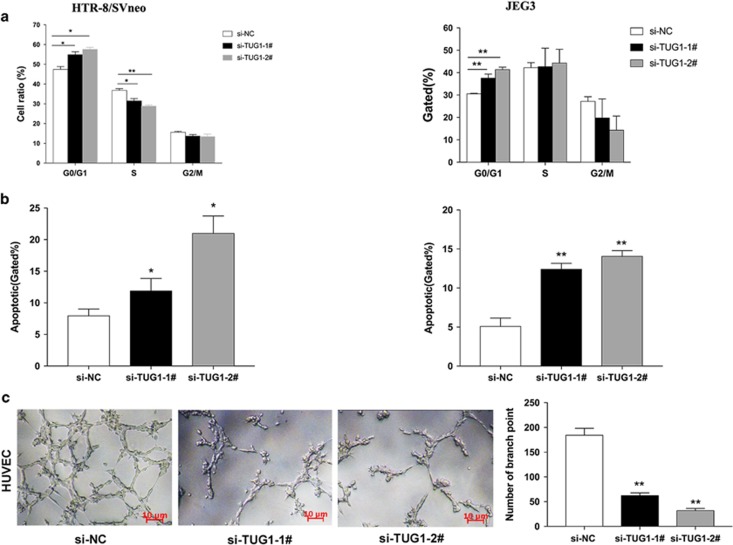
Effect of *TUG1* on cycle, apoptosis and network formation *in vitro*. Trophoblast Cells were treated with specific *TUG1* siRNAs. (**a**) Cell cycle analyses by Flow cytometry in HTR-8/SVneo and JEG-3 cells. (**b**) Flow cytometry was used to detect the apoptotic rates of cells. LR, early apoptotic cells; UR, terminal apoptotic cells (values are mean±S.E.M.; **P<0.01, *P<0.05). (**c**) Performing network formation, cells transfected with siRNAs targeting *TUG1* showed an increase in node numbers as compared with the negative control. All experiments were performed in biological triplicates with three technical replicates (values are mean±S.E.M.; *P<0.05; **P<0.01)

To examine whether the effect of *TUG1* on cells proliferation reflects changes in the cell cycle, we performed flow cytometry to examine cell cycle progression. The results showed that two effective siRNA transfected cells caused cell accumulation in the G1/G0 phase and/or entered the S phase less often compared with the cells which treated with scramble siRNAs (Figure 3a). Additionally, flow cytometry assays were performed to establish whether the silencing of *TUG1* affected the cell apoptosis. The proportion of both early apoptotic and late apoptotic cells were increased following *TUG1* knockdown in cells (Figure 3b). These data imply that *TUG1* promotes a proliferation phenotype in trophoblasts.

### Effects of *TUG1* on network formation ability *in vitro*

The failure of spiral artery remodeling can result in placental ischemia and hypoxia and further result in the occurrence and development of PE.^4, 17^ In this study, we explored the effect of *TUG1* on the network formation ability of associated cell lines *in vitro*, and further noticed the personality of *TUG1* in process of PE. As shown in Figure 3c, the number of capillary-like networks was significantly reduced after transfection of *TUG1* siRNAs and further decreased network formation ability.

### Gene expression profiling

**Figure 4:**
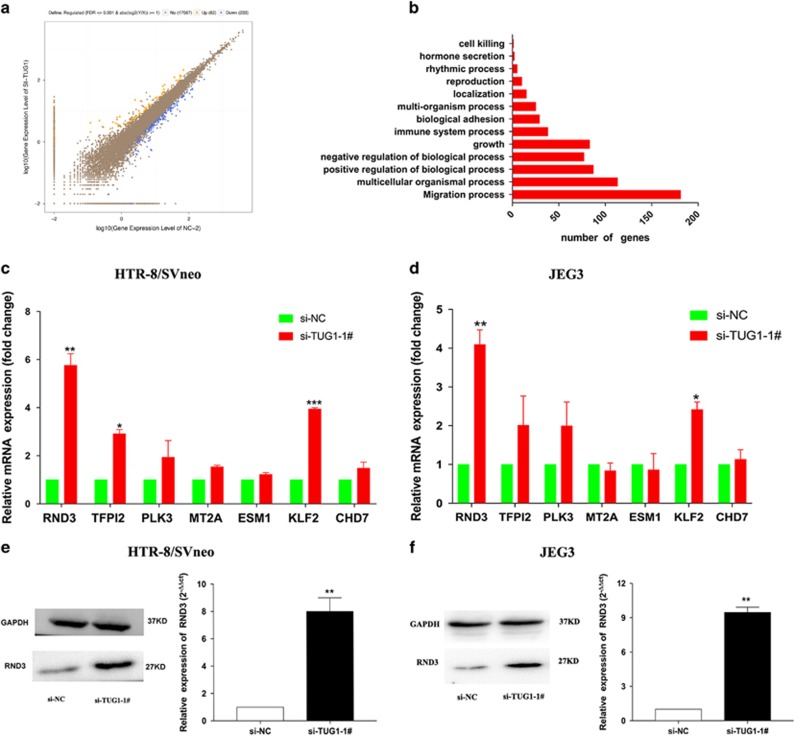
*TUG1* knockdown increases the expression of genes involved in cell proliferation and migration. (**a**) RNA transcriptome sequencing analysis was performed to analyze gene expression profiling in HTR-8/SVneo cells following *TUG1* knockdown. The picture showed the all of different expressed gene. (**b**) GO analysis for all genes with altered expressions between the scrambled siRNA-treated and si-*TUG1*-treated cells *in vitro*. Cell growth was among the significant biological processes for genes whose transcripts level were changed in the *TUG1*-depleted Trophoblast cells. (**c**,**d**) qRT-PCR analysis in si-*TUG1*-treated trophoblast cells reveal altered mRNA level of genes involved in cell proliferation and migration upon *TUG1* depletion. (**e**,**f**) Western blotting assays of RND3 protein levels after si-*TUG1* or si-NC was transfected into HTR-8/SVneo and JEG-3 cells. *GAPDH* protein was used as an internal control. Values represent the mean±S.E.M. from three independent experiments (values are mean±S.E.M.; **P<0.01, *P<0.05)

To determine *TUG1*-associated transcriptional changes, we performed RNA transcriptome sequencing of the control and si-*TUG1*-1#-treated HTR/SVneo cells to reveal latent downstream targets. The cell lines were treated with scramble and si-*TUG1*-1# for 36 h. Based on the RNA transcriptome sequencing data, the transcript levels of 82 genes exhibited ≥2-fold increases in abundance in HTR/SVneo cells after *TUG1* knockdown, while 233 genes exhibited ≤2-fold decreases in abundance (Figures 4a and b; Supplementary tables 2 and 3).

The assessment of pathways activated by *TUG1* according to the GO and KEGG databases indicated that cell growth, apoptosis and migration were altered in *TUG1*-depleted cells (Figure 4c). Noticeably, the GO results were fundamentally in accordance with our previous experimental findings. Using qRT-PCR, we confirmed the changes in gene expression that particularly affected the growth of HTR-8/SVneo and JEG-3 cells. Our results showed that *RND3* was significantly upregulated after *TUG1* knockdown (Figures 4c and d), indicating that the dysregulated genes *RND3* may be a crucial downstream mediator of *TUG1*. Western blotting assay was also performed and further confirmed that the Rnd3 protein levels were significantly increased in *TUG1*-depleted cells (Figures 4e and f).

### *TUG1* epigenetically silences *RND3* transcription by interacting with EZH2

**Figure 5:**
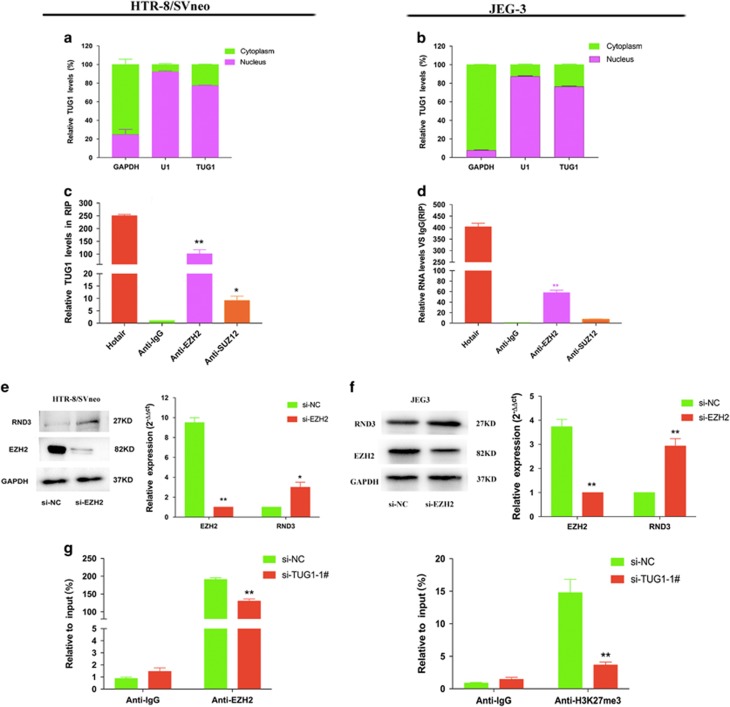
*TUG1* can recruit EZH2 to silence *RND3* expression. (**a**,**b**) Cell fractionation assays indicated that *TUG1* is mostly located in nucleus. GAPDH and U1 acted as the marker of cytoplasm and nucleus, respectively. (**c**,**d**) RIP assays established that *TUG1* can recruit *EZH2*. (**e**,**f**) The silencing of *EZH2* increased *RND3* expression at protein levels. (**g**) The enrichment of *EZH2* and H3K27me3 in the promoter regions of *RND3* were identified via ChIP assays, and this enrichment was decreased after *TUG1* knockdown. Values represent the mean±S.E.M from three independent experiments (values are mean±S.E.M.; **P<0.01, *P<0.05)

LncRNAs participate in regulating cell function by inactivating suppressors or activating enhancing factors via interactions with specific RNA-binding proteins (RBPs). To explore the potential biological mechanism of *TUG1* activity in trophoblast cells, we performed a molecular/cytoplasm separation, in which *GAPDH* was used as a cytoplasmic biomarkers and U1 acted as a biomarker of the nucleus. We then detected the subcellular localization of *TUG1*, which may supply cues for its role in molecular processes in trophoblast cells. The results showed that *TUG1* was essentially located in the cell nucleus in HTR-8/SVneo and JEG-3 cells (Figures 5a and b). Moreover, FISH assays were implemented to visualize the expression and subcellular localization of *TUG1* in HTR-8/SVneo cells (Supplementary Figure 1b). The data indicated that *TUG1* localized in the cytoplasm as well as the nucleus, but that levels of *TUG1* were higher than in the cytoplasm.

Previous studies have reported that lncRNAs can interact with RBPs to exert their regulatory functions. We therefore determined the interaction probabilities of *TUG1* with different RBPs through RNA–protein interaction prediction (http://pridb.gdcb.iastate.edu/RPISeq/) and discovered that *TUG1* potentially bind with Ezh2 and Lsd1 (RF or SVM score >0.5). Accordingly, we hypothesized that *TUG1* may regulate *RND3* expression by recruiting particular RBPs in trophoblast cells. Next, we conducted RNA immunoprecipitation (RIP) assay and found that *TUG1* can recruit Ezh2 in HTR-8/SVneo and JEG-3 cells (Figures 5c and d). Together, these data confirm the interaction between Ezh2 and *TUG1.*

Based to the RIP results, we also evaluated the relational features of the correlation between *TUG1* and Ezh2. Following the knockdown of *EZH2* by specific siRNAs, we found significant upregulation of *RND3* expression (Figures 5e and f). Many studies have established that Ezh2 acts as a suppressor of RNA transcription through histone modification, namely, H3K27me3. Hence, it is possible that *TUG1* represses the expression of *RND3* through recruiting Ezh2 protein to the promoter regions of *RND3*, mediating H3K27me3. Next, we performed ChIP assays and revealed that Ezh2 could directly interact with *RND3* promoter regions, leading to the trimethylation of H3K27 at gene promoter regions (Figure 5g). These results indicate that *TUG1* can promote trophoblast cells proliferation, apoptosis, and migration partially by repressing *RND3* expression.

### *RND3* expression levels are elevated in PE placental tissues and knockdown *RND3* is potentially involved in the biological role of *TUG1*

**Figure 6:**
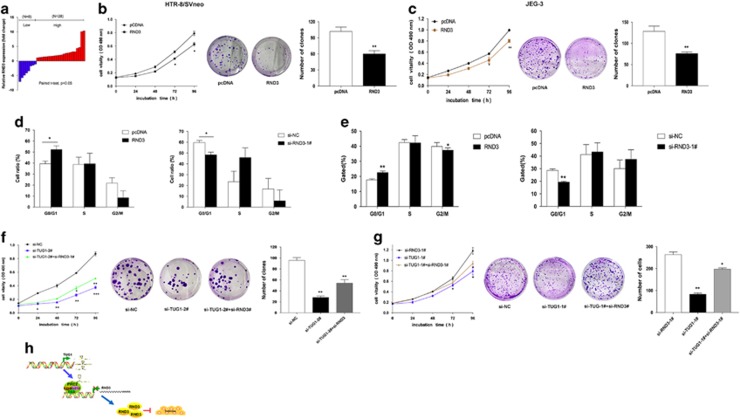
Upregulation of *RND3* inhibit trophoblast cell proliferation and were involved in the function of *TUG1*. (**a**) *RND3* expression levels were presented as the fold-change in PE placental tissue samples compare to the control. (**b**,**c**) MTT assays were executed used to determine the cell viability for pcDNA3.1-*RND3*-treated trophoblast cells. Colony formation assays were performed to assess the cell proliferation for pcDNA3.1-*RND3*-treatted HTR-8/SVneo and JEG-3. (**d**,**e**) Cell cycle analyses were utilized to examine after transfecting with siRNAs and/or pcDNA3.1-*RND3* in trophoblast cells. (**f**,**g**) MTT assays and colony formation assays were implemented to assess the cell viability for si-*TUG1* and si-*RND3* co-transfected trophoblast cells. (**h**) Summary diagram describes that *RND3* regulates trophoblast cell proliferation. Experiments were performed three times independently (values are mean±S.E.M.; **P<0.01, *P<0.05)

According to the above discoveries, we assessed whether *TUG1* regulates *RND3* expression in patients with PE. For this purpose, we evaluated the expression of *RND3* mRNA in 28 patients with PE. qRT-PCR analysis showed that *RND3* mRNA expression was elevated in placental samples from PE patients compared with levels in controls (Figure 6a).

To confirm whether *RND3* is involved in the biological function of *TUG1*, we performed gain- function assays in trophoblast cells. By qRT-PCR, *RND3* was significantly upregulated in trophoblast cells treated with GFP-*RND3*. In contrast, *RND3* was meaningfully downregulated in HTR-8/SVneo and JEG-3 cells after effective transfection with *RND3* effective siRNAs, respectively. Proliferation assays implied that the overexpression of *RND3* inhibited cell growth in HTR-8/SVneo and JEG-3 cells (Figures 6b and c). Next, flow cytometry analysis showed that upregulation of *RND3* increased G0/G1 phase accumulation, while the downregulation of *RND3* reduced G1/G0 phase arrest (Figures 6d and e). These results showed that *RND3* inhibition promotes cell cycle progression and proliferation of HTR-8/SVneo and JEG-3 cells.

To investigate whether *RND3* was involved in *TUG1*-induced HTR-8/SVneo and JEG-3 cell growth, we performed rescue experiments. These two cells were co-transfected with *TUG1* and *RND3* siRNAs. MTT and colony formation assays were conducted, and indicated that co-transfection moderately rescued si-*TUG1*-induced proliferation of HTR-8/SVneo and JEG-3 cells (Figures 6f and g). These results revealed that *TUG1* can promote trophoblast cell proliferation partially by silencing of *RND3 in vitro*.

Taken together, our findings confirm that *TUG1* binds to Ezh2 and epigenetically inhibit *RND3* expression in PE by mediating H3K27 trimethylation in the *RND3* promoter regions.

## Discussion

Recently, increasing researches has indicated that lncRNAs may have pivotal roles in cellular development and progression of multiple diseases,^18, 19^ including PE.^20, 21, 22^ Our previous findings also revealed that the abnormal expression of the lncRNAs *SPRY4-IT1* and *MEG3* can affect the cellular growth, apoptosis, migration, and invasion of trophoblast cells^22, 21^ to induce the occurrence and development of PE.

In this study, we focused on another lncRNA, *TUG1*, which is significantly downregulated in the placental samples of PE patients compared with that levels in controls, indicating that decreased *TUG1* levels might be associated with PE development and/or progression. Here, we performed relevant assays and confirmed that the silencing of *TUG1* expression exerts a suppressive effect on trophoblasts by inhibiting cell proliferation, invasion, and migration and promoting cell apoptosis. To our knowledge, the endometrial epithelial cells may act upon the trophoblast cells to stimulate the essential cellular steps for the trophoblast transformation of the spiral arteries. A disorder of spiral artery remodeling may lead to the pathogenesis of PE. Then, we executed network formation assays. According to network formation assays (Figure 3c), the tube formation after knockdown of *TUG1* were reduced compared with than that in controls. These resulting data imply that the aberrant expression of *TUG1* may trigger implications for poor transformation of maternal spiral arteries, contributing to placental abnormalities or PE. Previous studies have reported that *TUG1* could affect multiple cell biological functions, such as the normal formation of photoreceptors in the developing retina of rodents^23^and the growth, proliferation and invasion, and apoptosis of oral squamous cell carcinoma.^24, 25, 26, 27^ Thus, we hypothesis that *TUG1* may have an essential role on the behavior of the trophoblast cells. Because the molecular mechanism remains unclear, we need to further exploration, and screening of valuable biomarkers and therapeutic targets of PE are needed.

To explore the downstream effects of *TUG1* in PE, we performed RNA transcriptome sequencing to reveal latent downstream targets of *TUG1*. We found a noticeable increase in the expression of the cell suppressor Rnd3. Rnd3, also known as RhoE, is a GTPase that involve in the regulation of various cell biological behaviors including proliferation, migration, invasion, and apoptosis.^28, 29, 30, 31, 32^ Riento *et al.*^28^ has reported that Rnd3 is a suppressor of Rock1, which modulates actin dynamics. Previous studies have demonstrated the function of Rnd3 in cancer occurrence and progression.^33, 34^ For example, Rnd3 is significantly downregulated in lung cancer cells and can regulate lung cancer cell proliferation through Notch signaling.^35^ However, the function of Rnd3 in PE has not been studied. In the present study, we also found that the levels of *RND3* mRNA and proteins can be repressed by *TUG1* and that Rnd3 might act as a suppressor in trophoblasts.

Our research has confirmed EZH2 as an essential regulator in the *TUG1*-mediated Rnd3 repression network. qRT-PCR and western blot assays established that *RND3* were upregulated after the silencing of EZH2 in trophoblast cells. In present research, we revealed high abundance interacting between *TUG1* and EZH2 in trophoblast cells, and established that *TUG1* could mediate transcriptional regulation of *RND3*. ChIP-qPCR assays confirmed that *TUG1* can recruit Ezh2 protein and downregulated *RND3* expression. Our resulting data clarified that *RND3* expression is explicitly regulated by PRC2, partly through *TUG1*. Together, this demonstrates that *TUG1* affects the function of trophoblasts partly via the epigenetic regulation of *RND3* expression.

In short, our research showed that the lncRNA *TUG1* is downregulated in placental samples from PE patients compared with levels in controls. Knockdown of *TUG1* resulted in suppressive effects, impairing cellular proliferation, migration, and invasion and inducing apoptosis in trophoblasts, and further affecting the process of spiral artery remodeling. Based on these collective results presented above, we propose that *TUG1* bring into play functions, partially by recruiting Ezh2 and inhibiting *RND3* expression in PE (Figure 6h).

## Materials and methods

### Tissue samples and patients

We collected 52 paired placental tissues from PE women and normal pregnancies, who were diagnosed with PE and underwent cesarean deliveries at the Jiangsu Province Hospital in 2015–2016, then all placental tissues were instantly snap frozen with liquid nitrogen and collected at −84 °C before RNA extraction. And all patients’ clinic features were recorded in Table 1. Our research was authorized by the Ethics Committee of the First Affiliated Hospital of Nanjing Medical University, China. Informed written consents were gotten from all patients in this study.

### Cell culture

We selected three cell lines that were related to pregnancy. HTR-8/SVneo Cells was generous furnished by Prof. Charies Graham, Queen’s University, Canada. And JEG-3, HUVEC-C, WISH and BeWo cells were obtained from the Type Culture Collection of the Chinese Academy of Sciences (Shanghai, China). HTR/SVneo, JEG-3, BeWo, WISH and HUVEC-C cell lines were cultured in RPMI 1640, MEM, F12K, RPMI 1640 and ECM (KeyGEN, Nanjing, China), respectively, which added to 10% fetal bovine serum (GIBCO, BRL, Invitrogen, Carisbad, CA, USA),100 U/ml penicillin and 100 mg/ml streptomycin (Invitrogen) in humidified air at 37 °C/5% CO_**2**_.

### Cell transfection

Plasmid vectors (pcDNA-RND3 and empty vector) for transfection were arranged by using DNA Midiprep kits (Qiagen, Hilden, Germany). The specific siRNAs were transfected into cell lines, including si-*TUG1*, si-*RND3* or si-NC. The siRNAs sequences were presented in Supplementary Table 1. These cell lines were cultivated on six-well plates and then treated through using Lipofectamine-2000 (Invitrogen) following the manual. After 48 h transfection, cells were harvested for conducting further experiments.

### RNA preparation and qRT-PCR

RNA preparation and qRT-PCR assays were performed as previously reported in Zuo *et al.*^20^ 1 *μ*g RNA was reverse transcribed in a final volume of 20 *μ*l by using the PrimeScript RT reagent Kit (TaKaRa, Dalian, China). SYBR Premix Ex Taq (TaKaRa, Dalian, China) were used to examine the expression of *TUG1*, according to the manual. All results were normalized to GAPDH. The relative primers sequences were presented in Supplementary Table 1. The qPCR results were evaluated and afterwards converted to fold changes.

### Subcellular fractionation location

Subcellular fractionation location assays were performed as previously reported in Zuo *et al.*^20^ We used PARIS Kit (Life Technologies, Carlsbad, CA, USA) to separate of nuclear and cytosolic fractions of trophoblast cells according to the manual. The levels of *TUG1*, RNA *U1*and *GAPDH* in cytoplasm and nuclear fraction was detected by qPCR. The relative rate of *TUG1*, *GAPDH* and *U1* in cytoplasm or nuclear part was presented as the percentage of the total RNA. *U1* was used as nuclear control, and *GAPDH* as cytoplasm control.

### Fluorescence *in situ* hybridization

Cells were cultivated in 24-well plates, and then fixed by 75% ethanol for 30 min followed by washes with PBS. Next, the fixed cells were further conducted assays, according to the manual. The sequences of probe were listed in Supplementary Table 1.

### Cell viability assays

Cell proliferation assays, colony formation assays were performed as previously reported in Zuo *et al.*^20^

For EdU assays, cells were cultivated in six-well plates, and 10 *μ*M EdU was added to these wells. And then fixed with 4% formaldehyde for 30–40 min after 2 h. After washing, EdU could be identified with a Click-iTR EdU Kit for 25 min, and the cells were stained with 300 *μ*l DAPI (Invitrogen, Molecular Probes, Eugene, OR, USA) at 25 °C for 15–20 min and imagined by performing a fluorescent microscope (Nikon Corporation, Tokyo, Japan). The relative percentage of Edu-positive cells were examined from three to five subjects in three wells.

### Flow-cytometric analysis

Flow cytometry assays were performed as previously reported in Zuo *et al.*^20^ After the cells were transfected with siRNAs for 48 h, we harvested the cells and then performed FITC-Annexin V and Propidium iodide (PI) by using the FITC-Annexin V Apoptosis Detection Kit (BD Biosciences, Franklin Lakes, NJ, USA) according to the manual. Cell cycle analysis were stained with propidium oxide by the Cycle TEST PLUS DNA Reagent Kit (BD Biosciences) following the manual and evaluated by FACScan. The rate of the cells in each phase were assessed.

### Cell migration and invasion assays

Cell migration and invasion assays were performed as previously reported in Zuo *et al.*^20^ 3–5 × 10^4^ cells were cultivated on the top of a membrane precoated with Matrigel (BD Biosciences) for cell invasion assays (without Matrigel for cell migration assays). Cells inside the upper chamber were removed after incubation for 24, 36, 48 and 60 h. While cells on the lower membrane surface were fixed with methanol and then stained with 0.5% Crystal violet solution. Three-five randomly selected fields were counted in per well.

### Network formation assay

After 36 h of treated HTR-8/SVneo and HUVEC-C cells with *TUG1* siRNAs, then perform Network Formation assays as previously reported in Zou *et al.*^22^ The HTR-8/SVneo and HUVEC-C cells were transfected with siRNAs (10^4^ cells/well) and then cultivated in 96-well plates with five duplicate. After 6 h, the images were blinded and the number of cell–cell protracted contacts was counted as representations of the number of capillary-like networks present in each field. Results were presented as the average number of networks at least three separate experiments.

### Western blotting assays

HTR-8/SVneo and JEG-3 cells were harvested, and protein extractions was separated from transfected cells by using 10% polyacrylamide gradient SDS gel, and incubated with different antibodies, in which Anti-*GAPDH* and anti-*EZH2* were purchased from Abcam (Hong Kong, China). Anti-*RND3* was from Cell Signaling Technology (Boston, MA, USA).

### RNA-seq bioinformatic analysis

The mRNA-Seq experiments were executed by Wuhan Genomics Institute (Wuhan, China). mRNA-seq library was settled for sequencing exploiting standard Illumina protocols. Briefly, total RNAs from si-NC, or si-*TUG1* 1# transfected HTR-8/SVneo, were isolated by using TRIzol reagent (Invitrogen). mRNA extraction was performed by utilizing Dynabeadsoligo (dT) (Invitrogen Dynal). Superscript II reverse transcriptase (Invitrogen) and random hexamer primers were utilized to synthesize double-stranded complementary DNAs. To stablish the mRNA-seq library, the cDNAs were next fragmented via nebulization and the standard Illumina protocol followed.

### RNA immunoprecipitation assays

RNA immunoprecipitation assays were performed as previously reported in Zuo *et al*,^20^ HTR-8/SVneo and JEG-3 cells were lysed for immunoprecipitation of *EZH2* and *SUZ12*. The supernatants were incubated with protein A/G Sepharose beads coated with antibodies for 8–10 h at 4 °C. After the beads were washed with corresponding wash buffer, the complexes were incubated with 0.1% SDS/0.5 mg/ml Proteinase K (30 min at 55 °C) to remove proteins, respectively. The RNA was subjected by qPCR analysis to demonstrate the presence of *TUG1.*

### Chromatin immunoprecipitation assays

Trophoblast cells were preserved with formaldehyde and incubated for 10 min to generate DNA–protein cross-links. Then cell lysates were sonicated to generate chromatin fragments of 200–300 bp and immunoprecipitated with *EZH2* (Millipore, Billerica, MA, USA), H3K27me3 (Millipore) or IgG as the control. Precipitated chromatin DNA was recovered and analyzed by qRT-PCR.

### Statistical analysis

All statistical analyses were executed utilizing SPSS 17.0 software (IBM, Chicago, IL, USA). Furthermore, less than 0.05 considered statistically significant *P*-values. These resulting data were recounted as the mean±SD. Statistical significance were ascribed at *P<0.05 or **P<0.01.

## Figures and Tables

**Table 1:** Clinical characteristics of normal and pre-eclamptic pregnancies

**Variable**	**PE (*N*=52)**	**Control (*N*=52)**	***P*****-value**^**a**^ **normal *versus* *P***
Maternal age (year)	31.23±5.035	32.33±4.013	>0.05
Maternal weight (kg)	70.28±10.94	69.99±8.036	>0.05
Smoking	0	0	>0.05
Systolic blood pressure (mm Hg)	159.65±16.763	116.39±9.112	<0.01
Diastolic blood pressure (mm Hg)	103.11±16.763	74.12±8.676	<0.01
Proteinuria (g/day)	>0.3	<0.3	<0.05
Body weight of infant (g)	2126.54±793.945	3383.43±514.442	<0.05

Abbreviation: PE, pre-eclampsia.
